# ModularBoost: an efficient network inference algorithm based on module decomposition

**DOI:** 10.1186/s12859-021-04074-y

**Published:** 2021-03-24

**Authors:** Xinyu Li, Wei Zhang, Jianming Zhang, Guang Li

**Affiliations:** State Key Laboratory of Industrial Control Technology, Institute of Cyber-Systems and Control, Zhejiang University, Zheda Road, 310027 Hangzhou, China

**Keywords:** Regulatory network inference, Gene module Decomposition, GRNBoost2, Linear regression

## Abstract

**Background:**

Given expression data, gene regulatory network(GRN) inference approaches try to determine regulatory relations. However, current inference methods ignore the inherent topological characters of GRN to some extent, leading to structures that lack clear biological explanation. To increase the biophysical meanings of inferred networks, this study performed data-driven module detection before network inference. Gene modules were identified by decomposition-based methods.

**Results:**

ICA-decomposition based module detection methods have been used to detect functional modules directly from transcriptomic data. Experiments about time-series expression, curated and scRNA-seq datasets suggested that the advantages of the proposed ModularBoost method over established methods, especially in the efficiency and accuracy. For scRNA-seq datasets, the ModularBoost method outperformed other candidate inference algorithms.

**Conclusions:**

As a complicated task, GRN inference can be decomposed into several tasks of reduced complexity. Using identified gene modules as topological constraints, the initial inference problem can be accomplished by inferring intra-modular and inter-modular interactions respectively. Experimental outcomes suggest that the proposed ModularBoost method can improve the accuracy and efficiency of inference algorithms by introducing topological constraints.

## Background

In recent years, systems biology has developed rapidly. With the continuous development of high-throughput analysis technologies such as proteomics and transcriptomics [1], it has become possible to infer gene regulatory networks (GRNs). The main purpose of GRN inference is to determine causal relations between genes. Such networks offer important information about regulation and boost people’s understanding about mechanisms.

After several decades of development, computational efficiency and accuracy of network inference algorithms have increased dramatically. Large gene networks of micro-organisms and mammals can be reconstructed using transcriptomics datasets. Methods with high accuracy in inferring GRNs have been proposed. For example, the TIGRESS [2] and fused LASSO [3] based on linear regression have exhibited superior performance in computational efficiency, while machine learning-based methods such as GENIE3 [4] and GRNBoost2 [5, 6] in boosting framework are widely used due to their advantages in accuracy. Mutual information-based CLR [7] and PIDC [8] can reveal the statistical dependencies among genes.

However, there are some limitations also exist in those GRNs inference methods. Inferred topologies usually lack clear biophysical explanations, limiting their applications such as disease-gene prediction and gene therapy. For GRN and protein–protein interaction (PPI) networks, a key character shared by biological networks is the so-called functional module or communities structure [9]. Each module corresponds to a sub-network in which nodes are densely connected and exchange information frequently [10]. Besides, traditional approaches determine the casual relations between genes at a single stage, leading to considerable computational burden. Parameter estimation of GRN with topological constraints had exhibited advantages in computational efficiency [11]. According to the regulatory module theory, inter-modular connections have a more tight association than the genes pairs in intra-module [12]. When the conventional inference task can be accomplished at multiple stages, the efficiency of network inference may be expected to be improved.

With accurately detected modules, it is feasible to develop an efficient inference framework that combines inherent modular structures with established inference algorithm. Plenty of module identification methods have been developed to detect functional modules from GRN, PPI, and other biological networks. For gene module detection approaches including CoReg and SigMod etc[13, 14], network topologies were required to be known. This is a strong prerequisite that is hard to be satisfied in real applications. In this context, data-driven module identification methods become crucial to identify modules directly from transcriptomic data. Decomposition-based and clustering-based methods have attracted increasing attention due to the ability to detect gene modules from transcriptomic data [15]. Although gene modules can be detected, current researches focus on finding the biological explanations and relevant pathways to some extent [16]. From the viewpoint of network inference (NI), gene modules provide a constraint to guide the inference, leading to GRN with community structures.

Motivated by these topological characters, this study proposes a ModularBoost method to integrate decomposition-based module identification and boosting-based inference algorithm. Using ICA-FDR, ModularBoost assigned genes to regulatory modules according to their expression data. Based on the detected gene modules, casual relations within gene modules were inferred by GRNBoost2 that is a top-ranking inference algorithm, while the regulatory relationships between modules were detected by linear sparse regression. Then ModularBoost normalized the scores from subnetworks to obtain the final network.

Among decomposition-based methods, ICA-FDR, ICA-zscore, and PCA have been implemented and compared with commonly-used clustering expression datasets [9]. And we selected the ICA-FDR algorithm that demonstrates the highest accuracy in module identification. Besides, the performance of ModularBoost method was evaluated by single-cell expression and time-series data. The simulated scRNA-seq datasets are generated by BEELINE [17] and PIDC [8]. The three experimental scRNA-seq data sets are from the SCODE project [18]. And the time-series data sets are the *S. aureus*, *E. coli* and Yeast expression data from the Dream5 challenge [19]. As for the gold standard of gene modules, based on partly known gene regulatory edges, functional modules were extracted by graph theory or community detection methods. This work not only discusses the applicability and accuracy of ModuleBoost in network inference (NI), but also further analyzes the relation between data-driven module detection and NI.

## Result

### Modular inference of simulated scRNA-seq datasets

Curated networks were extracted from Beeline project, which focused on GRN inference using single cell expression data. Different from traditional microarray datasets, single-cell data contains information about cell–cell variability and can be used to investigate behavior patterns of cell populations [20]. However, strong stochastic single cell expression data often lead to low accuracy in network inference. Even several algorithms including SCODE and PIDC have been developed [8, 18], the accuracy levels of GRN inference using single cell data were quietly low. Another bottleneck brought by single cell data was increasing computational burden, due to plenty of cell samples [21]. Cell–cell variability information in single cell expression data play a negative role in inferring TF-gene relations, leading to low accuracy of inference in many cases.

In this case, the proposed ModularBoost approach aims to improve the accuracy in inference by introducing topological constraints. In the proposed ModularBoost method, ICA-FDR based decomposition was used as inner part to detect functional modules directly from curated datasets. Competing methods include ICA-FDR2, ICA-zscore, PCA-based decomposition and K-means clustering methods. Performance index $F_{rr}$ using four decomposition methods and K-means clustering were described in this research. Curated datasets from the GSD network had three experimental conditions, depending on the dropout rates. PIDC *E. coli*-S denotes single-cell data with 700 cell samples, while both *E. coli*-LL and *E. coli*-LH represent datasets with 2000 samples. In addition, the *E. coli*-LL and *E. coli*-LH groups correspond to single-cell data with low and high dropout rates respectively. Evaluation metrics of the curated GSD network and PIDC *E. coli* network were compared in Table 1.

**Table 1:** Module identification evaluation of the curated GSD and PIDC *E. coli* network using $F_{rr}$

Methods	GSD-1	GSD-50	GSD-70	*E. coli*-S	*E. coli*-LL	*E. coli*-LH
ICA-FDR	**0.307**	**0.307**	0.268	**0.355**	0.345	**0.337**
ICA-FDR2	0.249	0.249	0.212	0.240	0.234	0.227
ICA-zscore	0.172	0.111	0.111	0.318	**0.359**	0.323
PCA decomposition	0.252	0.213	0.111	0.287	0.279	0.285
K-means	0.288	0.288	**0.288**	0.247	0.230	0.240

$F_{rr}$ values were positively related with the accuracy level in module detection. Highest values in each column were displayed in bold. ICA-FDR and ICA-FDR2 required the number of gene modules $n\_comp$ and the threshold of Q-value *q_cutoff* according to the number of genes in GRN. For the module identification evaluation of curated networks with 19 genes, we set consistently $n\_comp$ = 2 for five module identification methods. Meanwhile, PIDC *E. coli* network with 100 genes set $n\_comp$ = 4. And the *q_cutoff* of ICA-FDR, ICA-FDR2, and PCA was $10^{-3}$. However, the *q_cutoff* of ICA-zscore decomposition was different from ICA-FDR due to the difference in statistical principles: $\textit{q\_cutoff}\ ^{zscore}$ = 1.5. To eliminate the randomness of the heuristic algorithm, we repeated each gene network 10 times and took the average of $F_{rr}$.

A pattern that could be found in Table 1 was that ICA-FDR outperformed three decomposition methods and k-means clustering, showing high accuracy in module detection. Furthermore, $F_{rr}$ indexes obtained by ICA-FDR were slightly higher than that from ICA-FDR2. A possible explanation was that ICA-FDR2 algorithm had taken the direction of regulatory edges into consideration, thus influencing the accuracy of gene module identification. Meanwhile, dropout rate, which was regarded as noise, had shown negative impacts on module detection. $F_{rr}$ indexes from GSD-70 were lower than that from GSD-1 and GSD-50, using five module detection methods.

Instead of reconstructing a network as whole, the ModularBoost approach accomplished the GRN inference task based on the identified modules, which were densely connected genes and TFs. Furthermore, intra-modular and inter-modular interactions between genes were inferred in two stages of inference.

It can be observed from Table 2 that the proposed ModularBoost approach obtained highest AUROC indexes among selected inference algorithms including ridge and GRNBoost2 methods. Highest AUROC and AUPR in each column were displayed in bold. This phenomenon validates the effectiveness of ModularBoost as well as the integration of two inference strategies. The computational time of ModularBoost in the three curated networks and PIDC networks are also less than that of GRNBoost2. ModularBoost $time_{GSD-1} = 85$ s, $time_{GSD-50} = 73$ s, $time_{GSD-70} = 78$ s, GRNBoost2 $time_{GSD-1} = 119$ s, $time_{GSD-50} = 116$ s, $time_{GSD-70} = 100$ s. ModularBoost $time_{E. coli-S} = 16$ s, $time_{E. coli-LL} = 155$ s, $time_{E. coli-LH} = 137$ s, GRNBoost2 $time_{E. coli-S} = 67$ s, $time_{E. coli-LL} = 1362$ s, $time_{E. coli-LH} = 965$ s. The AUROC and AUPR indexes of GRNBoost2 and the proposed ModularBoost indicated that the ModularBoost approach was able to accomplish the inference task using reduced computational resource, without obvious loss of accuracy. From this perspective, the ModularBoost approach can be used as an efficient solution to infer TF-gene relations using single cell expression data. AUROC and AUPR indexes of GSD-70 group were lower than two other groups, suggesting negative impact of dropout rate. Especially when the dropout rate increase from 50 to 70, such impacts were obvious, both in module detection and network inference. Meanwhile, this situation was also confirmed at PIDC datasets. AUROC and AUPR indexes tend to decrease when the single-cell data sample sizes and dropout rates increase. In this study, dropout rate played the role of noise in single cell expression data and has a negative influence in GRN inference accuracy.

**Table 2:** AUROC and AUPR indexes for curated and PIDC networks with conventional GRN inference and ModularBoost

Methods	Curated GSD-1		Curated GSD-50		Curated GSD-70	
	AUROC	AUPR	AUROC	AUPR	AUROC	AUPR
Ridge	0.545	0.234	0.517	0.232	0.530	0.234
Linear regression	0.520	0.225	0.507	0.220	0.467	0.204
TIGRESS	0.547	0.233	0.548	0.253	0.523	0.249
GRNBoost2	0.547	0.230	**0.566**	0.258	0.550	0.258
ModularBoost	**0.549**	**0.234**	0.559	**0.260**	**0.553**	**0.259**

Methods	PIDC *E. coli*-S		PIDC *E. coli*-LL		PIDC *E. coli*-LH	
	AUROC	AUPR	AUROC	AUPR	AUROC	AUPR
Ridge	0.667	0.065	0.594	0.024	0.540	0.015
Linear regression	0.461	0.010	0.518	0.012	0.479	0.011
TIGRESS	0.676	0.068	0.612	0.035	0.601	0.020
GRNBoost2	0.659	0.056	0.558	0.038	0.570	**0.034**
ModularBoost	**0.678**	**0.074**	**0.624**	**0.039**	**0.618**	0.028

To show the superior network prediction of ModuleBoost2, we compared ModularBoost with the ridge regression based inference method from the perspective of degree distribution [22]. The degree distribution $p_k$ of GRNs follows approximately the power-law [23], and it is given by:

1 $$\begin{aligned} \log p_k = -\alpha \log k + c \end{aligned}$$

where *k* denotes degree, $\alpha$ represents scaling coefficient and c is a constant.

**Fig. 1:**
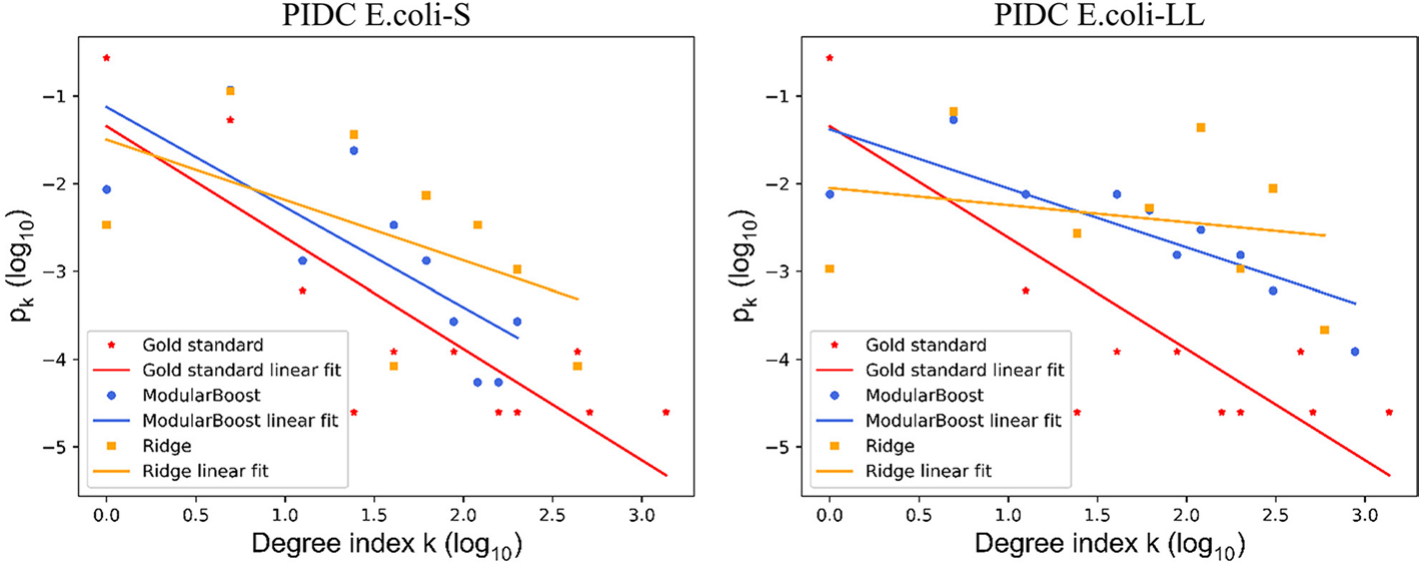
The power-law distributions of PIDC *E. coli*. Comparison of the power-law distribution and $\log _{10}$-$\log _{10}$ degree for gold standard network, ModularBoost-inferred network, and Ridge-inferred network of *E. coli*

The power-law distributions of PIDC *E. coli*-S and *E. coli*-LL were shown in Fig. 1. For simulated single cell expression datasets, Ecoli-S and Ecoli-LL share the same gold standard networks. These two cases may lead to different inferred structures, due to various complexity levels. When the sample size of expression data increases, the inference indexes tend to decline while the mismatch degree between inferred networks and gold standards has become more significant.

Under this circumstance, the proposed ModularBoost method can obtain GRNs that are higher topologically similar with gold standards, compared with ridge regression inference algorithm. In other words, ModularBoost is able to improve the interpretability of inferred networks to some degree.

### Modular inference of experimental scRNA-seq datasets

In this section, experimental single cell RNA sequencing(scRNA-seq) datasets were used as the major information source in GRN inference. In biomedical and genomic research, scRNA-seq datasets has played a crucial role in exploring dynamics of cell population and differentiation. Three scRNA-seq datasets and relevant gold standard networks were provided by the SCODE project. In Table 3, PrE (primitive endoderm cells), MEF (mouse embryonic fibroblast cells), DE (definitive endoderm cells) denote various cell types from mouse and human respectively. Regulatory edges in gold standard covered a subset of 100 genes, ranging from 40$\%$ to 60$\%$. Based on known regulatory edges, gene modules were detected using graph theory-based methods, i.e. community detections methods. Those modules were intersected, corresponding to overlapping phenomenon.

**Table 3:** The list of gene module gold standard for three SCODE networks

Network	module	Genes
PrE	module 1	XBP1 GATA6 MYBL2 RHOX6 JUN
	module 2	ETS1 EGR1 POU5F1 ELF3
	module 3	DNMT3A SIX1 SMAD7
	module 4	EPAS1 BHLHE40 POU5F1
MEF	module 1	HMGA2 ASCL1 FOS ATF3 ETS1 PRRX1 JUN NFIC FOSB
	module 2	FOS EBF1 PLAGL1 FOSB SOX9 KLF4
	module 3	NFIC NFIA NR2F2
DE	module 1	CDX1 JUND
	module 2	PRDM1 HAND1 FOXF1 HOXB6 SOX5 SOX17 TCF7 MAF
	module 3	OTX2 NFIB ZIC3 CDX1 LEF1 ZFX TFAP2A ZEB1
	module 4	GATA6 T EOMES GATA4 SOX2 MSX1 PITX2 POU5F1 NANOG
	module 5	T CDX1 JUND ZFP42 ARID3A GATA6 HAND1 ZFX

The basic idea of module standards is to explore strongly interconnected components where nodes are mutually connected. In module 2 of PrE network, ETS1, EGR1 and SMAD7 formed a connected component. ETS1 and EGR1 are hub nodes due to high out-degrees. In known regulatory relations, the SMAD7 gene owns 12 out-degrees and 16 in-degrees, indicating that this gene play a bridge node in the PrE network.

For the MEF network, those TFs such as KLF4 had high out-degrees and were believed to have high topological importance in information flows. According to known modules, overlapping existed, allowing part of genes belong to two or more functional modules.

**Fig. 2:**
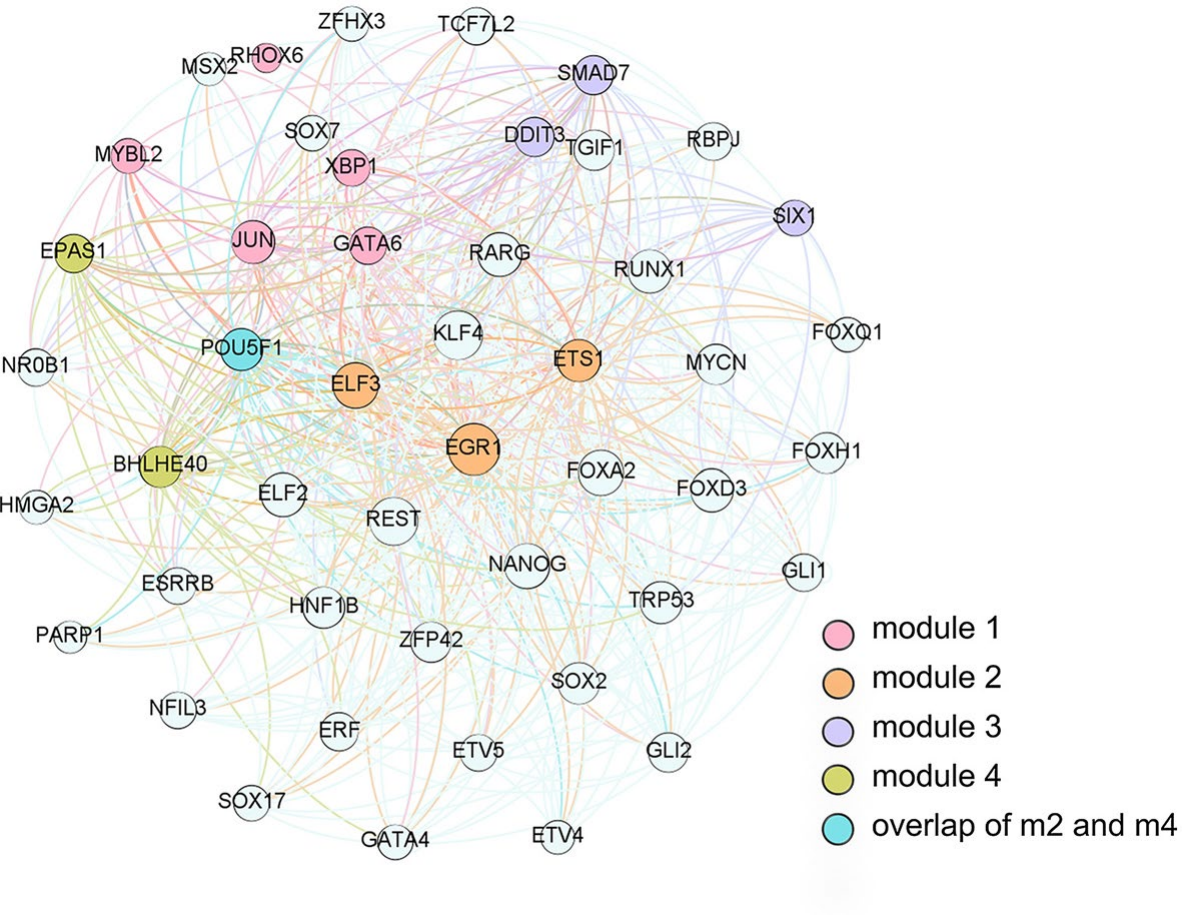
The gene module gold standard of SCODE PrE network. The overlap signifys POU5F1 was both involved in module 2 and module 4. The edges in the network represented the regulations between genes

In Fig. 2, gene modules were colored and visualized by the Gephi software to illustrate the topological positions. In the directed graphs, the physical sizes of nodes were positively related with their topological importance. Meanwhile, functional and topological neighborhoods were related but different. It can be observed from Fig. 2 that nodes in the same gene modules tend to densely connected and had topological relations. In this study, gene modules were determined by data-driven ICA-FDR method, leaving a part of genes uncolored.

For PrE, MEF, DE networks, the numbers of gene modules were assigned as 3, 4, 4, according to prior information.

**Table 4:** Comparison of module identification methods using experimental using scRNA-seq datasets

Methods	SCODE PrE	SCODE MEF	SCODE DE
ICA-FDR	**0.244**	0.215	**0.272**
ICA-FDR2	0.241	0.210	0.141
ICA-zscore	0.233	**0.257**	0.267
PCA decomposition	0.100	0.180	0.216
K-means	0.102	0.138	0.129

$F_{rr}$ indexes were computed by four decomposition based methods and K-means clustering

From Table 4, high $F_{rr}$ indexes with bold obtained by ICA-based decomposition suggested their advantages in detection accuracy, compared with PCA-decomposition and k-means clustering. And $F_{rr}$ indexes of ICA-FDR2 were lower than that of ICA-FDR. This pattern was consistent with that in microarray datasets from curated subsection.

Based on the detected modules, the proposed ModularBoost approach accomplished the network inference using two kinds of regression: ridge regression and ensemble inference algorithm. This study chose cutting-edge ensemble inference named GRNBoost2 to determine intra-modular casual interactions. Table 5 shows AUROC and AUPR indexes obtained by the ModularBoost method and three candidate inference algorithms. And highest AUROC and AUPR in each column were displayed in bold.

**Table 5:** AUROC and AUPR for SCODE with classical GRN inference and ModularBoost

Methods	SCODE PrE		SCODE MEF		SCODE DE	
	AUROC	AUPR	AUROC	AUPR	AUROC	AUPR
Ridge	0.529	0.076	0.502	0.074	0.496	0.041
Linear regression	0.524	0.072	0.516	0.076	**0.505**	0.041
TIGRESS	0.501	0.062	0.487	0.066	0.502	0.042
GRNBoost2	0.466	0.058	0.474	0.067	0.503	**0.046**
ModularBoost	**0.532**	**0.077**	**0.540**	**0.088**	0.503	0.044

SCODE PrE and MEF datasets are single-cell expression data measured from mouse cells, while SCODE DE dataset denotes expression data of human cell populations. For PrE and MEF datasets, the proposed ModularBoost method obtained improved inference performance with reduced computational cost (ModularBoost $time_{PrE} = 17s, time_{MEF} = 24s, time_{DE} = 11$ s, GRNBoost2 $time_{PrE} = 335s, time_{MEF} = 230s, time_{DE} = 539$ s).

**Fig. 3:**
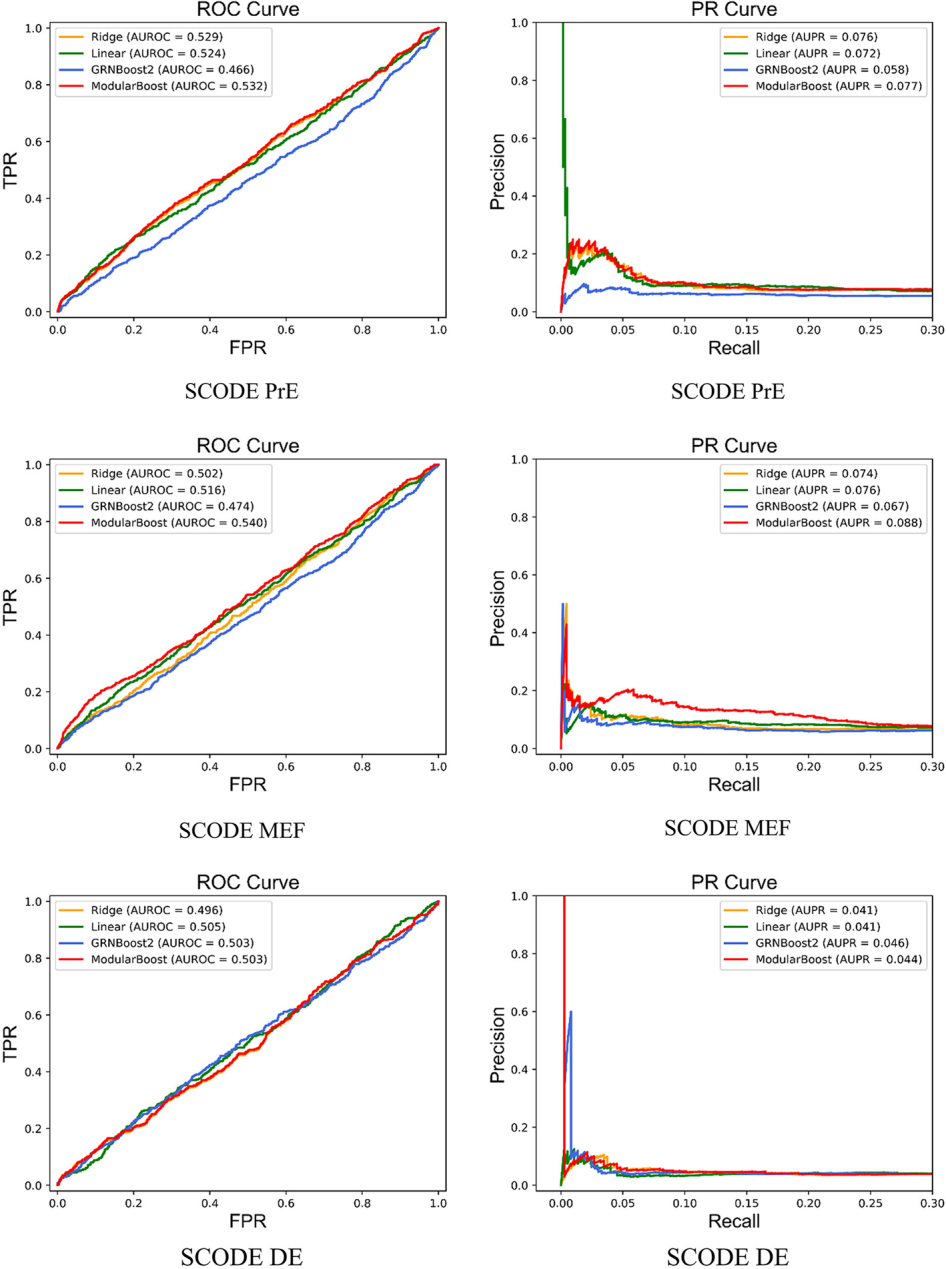
Performance on three SCODE networks. The area under ROC and PR is positively related with the accuracy in network inference

Figure 3 depicts both the PR and the ROC curves of four inference algorithms, during reconstruction of three SCODE networks. For PrE and MEF networks, the proposed ModularBoost approach outperforms three candidate inference methods in terms of AUPR and AUROC values. Moreover, the shape of the PR curve shows that the predicted lists inferred by ModularBoost method contains more true edges than other algorithms. For the DE dataset, the accuracy indexes of ModularBoost were lower than that of existing GRNBoost2. One possible explanation is the complex physiological process of gene regulation in human cell populations.

### Modular inference of DREAM5 networks

As a typical kind of time-series expression data, microarray datasets have long been used as information source in GRN inference. DREAM5 provided four networks as benchmarks for network inference research [19]. Considering the huge number of nodes and regulatory edges, the accuracy indexes obtained by existing methods were relatively low and the computational time was considerable. Among four DREAM5 networks, *S. aureus*, *E. coli* and Yeast networks had been used as benchmarks in this sections.

In DREAM5 challenge, ‘true’ regulatory relations were used as so-called gold standards to judge the accuracy degree of inference algorithms. Specially, the modular gold standard of DREAM5 was from Saelens [9]. As for the labels of gene modules, Sisima and Macisaac et. al provided two groups of incomplete module sets to quantitatively evaluate performance of candidate module identification methods [24]. Based on ICA-FDR, the ModularBoost approach firstly detected gene modules directly from DREAM5 datasets. The comparison of ICA-FDR with the other methods were shown in Table 6.

**Table 6:** Module identification evaluation of the DREAM5 networks

Methods	Module gold	DREAM5 *E. coli*	DREAM5 Yeast	
			Sisima	Macisaac
ICA-FDR	Minimal	**0.206**	**0.102**	**0.107**
	Strict	0.193	**0.090**	**0.073**
	Interconnected	**0.199**	**0.102**	**0.094**
ICA-FDR2	Minimal	0.203	0.093	0.099
	Strict	0.190	0.082	0.069
	Interconnected	0.189	0.094	0.087
ICA-zscore	Minimal	0.192	0.086	0.096
	Strict	**0.202**	0.070	0.064
	Interconnected	0.183	0.081	0.086
PCA decomposition	Minimal	0.101	0.047	0.047
	Strict	0.100	0.046	0.042
	Interconnected	0.097	0.047	0.048
K-means	Minimal	0.173	0.070	0.076
	Strict	0.178	0.059	0.055
	Interconnected	0.146	0.063	0.066

$F_{rr}$ indexes were calculated by comparing the predicted and known modules. High $F_{rr}$ values demonstrate high consistency between the predictions and module gold standards. Three types of gene modules, i.e. Minimal, Strict and Interconnected were taken into consideration. Highest values in each type of gene modules for networks were displayed in bold

Minimal, Strict, and Interconnected denote three definitions of gene modules, according to connectivity patterns. The Minimal modules can be regarded as the overlapping sets of genes that shared at least one TF. And Strict modules correspond to the gene sets that are regulated by the same regulators. For Interconnected modules, genes in the same module are strongly interconnect. For the same gold standard network, the Interconnected standard usually obtains the least number of gene modules.

For *E. coli* network with 4511 genes and Yeast network with 5950 genes , we set $n\_comp = [10, 20, \ldots , 100]$ and ${q_cutoff} = [10^{-1}, 10^{-2}, \ldots , 10^{-13}]$, leading to 130 parameter combinations. The parameter *q_cutoff* of ICA-zscore and PCA decomposition algorithms were settled as ${q\_cutoff}\ ^{zscore} = [0.5, 1, \ldots , 6.5]$, ${q\_cutoff}\ ^{pca} = [1, 0.75, 0.5, 0.25, 0.1, 0.075, 0.05, 0.025, 0.01, 0.0075, 0.005, 0.0025, 0.001]$. To reduce the stochastic impacts, decomposition-based module detection algorithms detected modules ten times for each parameter combination, and computed the average $F_{rr}$ indexes. In Table 6, the values $F_{rr}$ were obtained by taking the maximum value among 130 combinations.

**Fig. 4:**
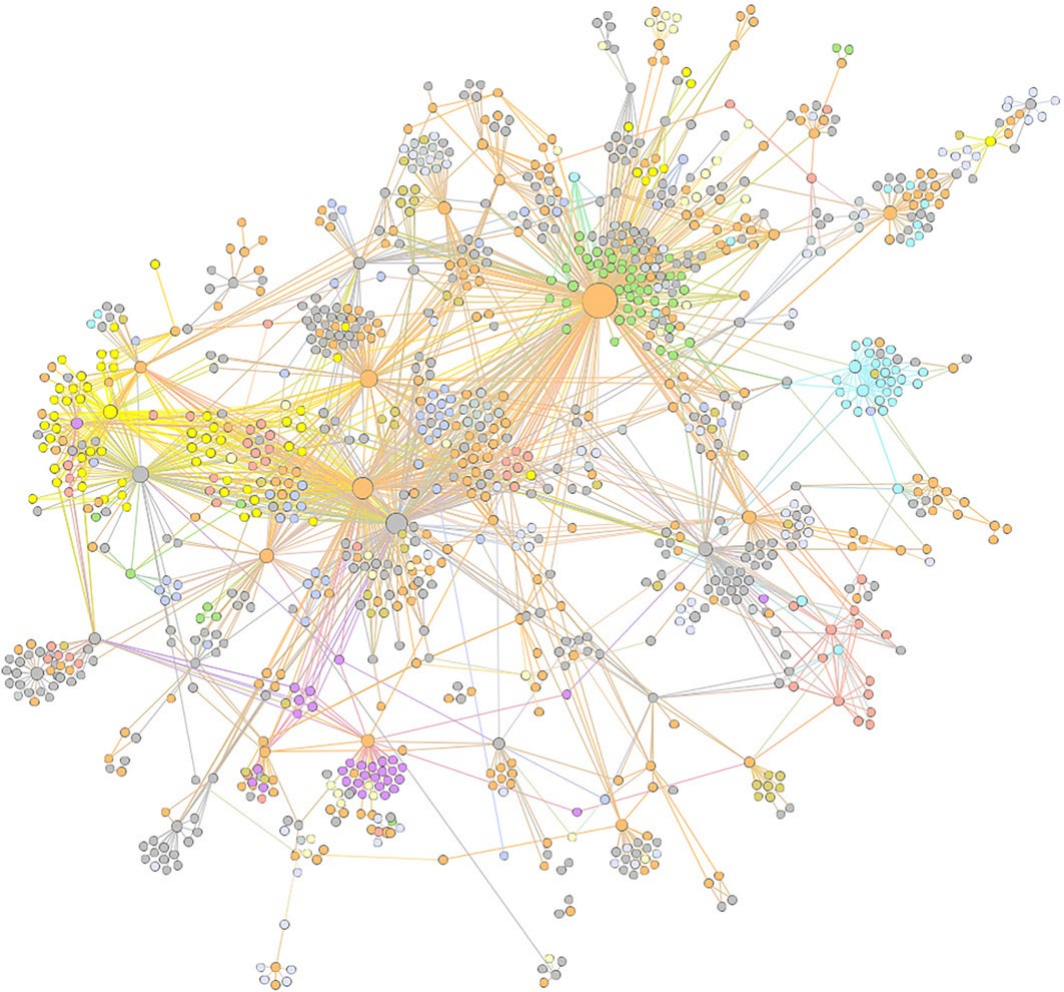
Module identification of  DREAM5 *E. coli* network by ModularBoost algorithm. The network topology was derived from the DREAM5 *E. coli* gold standard, and showed clear topological modules. Multiple gene modules were marked with different colors according to the decomposition results of ModualrBoost. ICA-FDR algorithm was employed in the decomposition part to detect functional modules from time-series expression data

It can be observed from Table 6 that decomposition-based approaches identify gene modules with enhanced accuracy than K-means method, under three module definitions. Among three module definitions, $F_{rr}$ indexes of the Minimal and Strict groups were higher than that of Interconnected criteria. This phenomenon indicated that Minimal co-regulation can be a suitable definition to evaluate module detection. To demonstrate the decomposition of ICA-FDR, regulatory modules were colored in DREAM5 *E. coli* gene networks shown in Fig. 4.

In Fig. 4, nodes in the graph represent TFs or genes and edges denote regulations. Those nodes colored with the same color were assigned to the same functional module, according to ICA-FDR decomposition in the ModularBoost method. With annotations, gene modules are crucial to deepen the understanding about regulation mechanisms with a given network. From this perspective, the ModularBoost method offered a data-driven solution to unveil functional modules directly from expression data, even without accurate annotations. Different from raw module identification, the ModularBoost method detects gene at the first stage, then infer inter-modular and intra-modular regulations at the second stage. Directed regulatory edges between TF-gene pairs will be necessary to further analyze information flow and potential biophysical explanations.

**Table 7:** Comparison of AUROC, AUPR indexes in DREAM5 networks

Methods	DREAM5 *S. aureus*		DREAM5 *E. coli*		DREAM5 Yeast	
	AUROC	AUPR	AUROC	AUPR	AUROC	AUPR
Ridge	0.667	0.004	0.610	0.067	0.529	0.019
Linear regression	0.657	0.004	0.606	0.056	0.530	0.019
TIGRESS	0.627	**0.021**	0.595	0.069	0.517	0.020
GRNBoost2	0.644	0.007	**0.642**	**0.101**	0.531	0.020
ModularBoost	**0.671**	0.010	0.624	0.069	**0.532**	**0.021**

In systems biology, densely connected nodes usually are related with specific cellular functions or diseases [25]. Under this circumstance, the ModularBoost method aims to provide reconstructed GRN topologies with clear community structures. This is an important character owned by the ModularBoost method. Other candidate inference algorithm including ridge regression based TIGRESS and ensemble-based GRNBoost2 majorly focus the whole network structure [2].

From the gene module detection outcomes of two DREAM5 networks, the ICA-FDR part in the ModularBoost method accomplished the first stage of task efficiently. In the subsequent inference of subnetworks, the AUROC and AUPR indexes were compared in Table 7. And the highest values in each column were marked in bold.

It can be observed from the Table 7 that the ModularBoost approach obtained higher AUROC and AUPR indexes than standard ridge and GRNBoost2 in two DREAM5 networks. The proposed ModularBoost approach integrates ensemble-based GRNBoost2 and ridge inference methods by introducing gene modules as topological constraints. The core of TIGRESS algorithm was regularized regression, leading to higher AUPR indexes than conventional linear regression methods. Network inference tasks were performed on a computer with 8 GB RAM, Intel i7-9750H 2.60 GHz. GRNBoost2 algorithm took 1 h 57 min to complete the inference of the DREAM5 *S. aureus* network, while ModularBoost only needed 7 min for the same task. GRNBoost2 algorithm took 41 h 46 min on DREAM5 *E. coli* network, while ModularBoost took 1 h 8 min, reducing approximately 96% computing efforts. The DREAM5 Yeast network took 33 h 36 min with GRNBoost2 inference, while only used 1 h 9 min with ModularBoost. Obviously, compared with GRNBoost2, ModularBoost significantly improved the speed of network inference, and did not cause a significant decrease in the accuracy of network inference. One advantage of introducing topological constraints is to improve inference accuracy. Another benefit is to reduce computational burden, especially for GRN with thousands of regulatory edges.

**Fig. 5:**
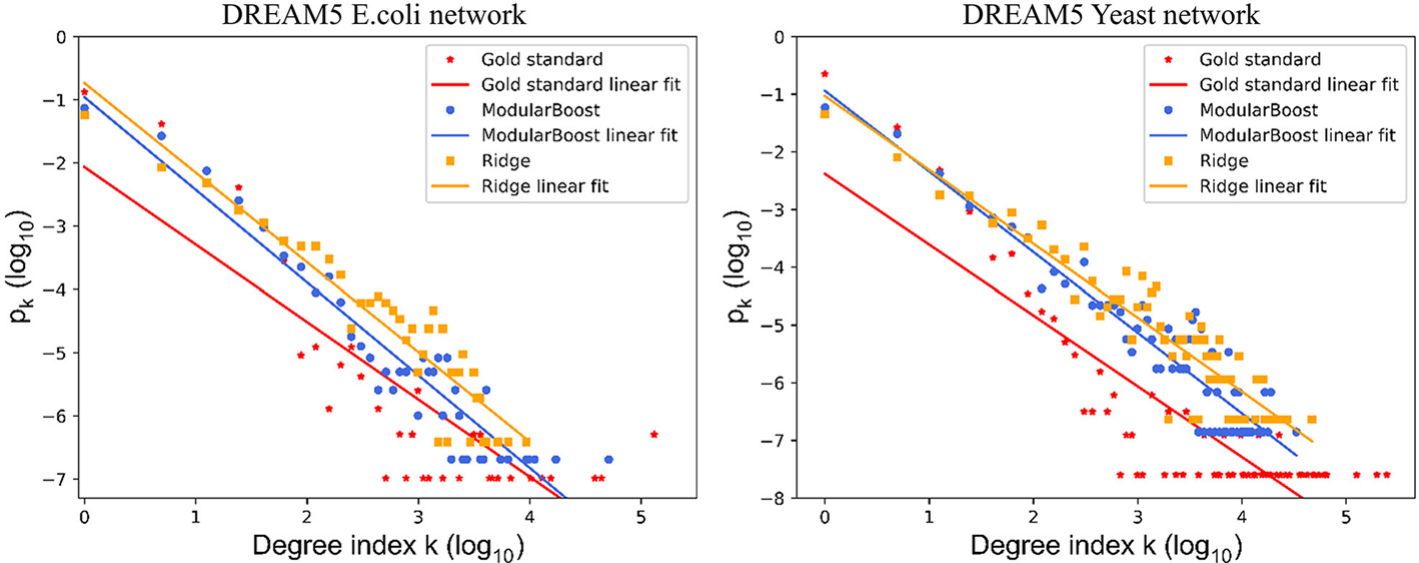
The power-law distributions of *E. coli* and Yeast. Comparison of the power-law distribution and $\log _{10}$-$\log _{10}$ degree for gold standard network, ModularBoost-inferred network, and Ridge-inferred network of *E. coli* and Yeast

For three types of GRNs, degree distributions were fitted by the power-law distribution, as shown in Fig. 5. Compared with ridge-based inference algorithm, GRNs obtained by the ModularBoost approach showed closer similarity with the gold standard networks. These results show that ModularBoost-inferred networks are more similar with the ‘true’ network topology. The linear fitting parameters are shown in Table 8.

**Table 8:** The linear fitting parameters $\alpha$ and *c* for power-law distribution

DREAM5	Network	$\alpha$	*c*
*E. coli*	Gold standard	1.225	$-$2.064
	ModularBoost	1.466	$-$0.956
	Ridge	1.418	$-$0.733
Yeast	Gold standard	1.226	$-$2.381
	ModularBoost	1.397	$-$0.940
	Ridge	1.280	$-$1.030

## Discussion and conclusions

This work aims to develop a seamless framework to perform GRN inference based on module identification. In order to detect modules from expression data, ICA-based decomposition algorithms have been applied in the proposed ModularBoost algorithm. Among several candidate decomposition methods, ICA-FDR had shown advantages in detection accuracy. In this case, ModularBoost employs the ICA-FDR algorithm to detect gene module from transcriptomic data. In the subsequent network inference part, intra-modular and inter-modular interactions were determined by ensemble-based and sparse regression-based algorithms respectively. The idea behind the ModularBoost is to introduce topological constraints to conventional network inference. Such topological constraints consider inherence community structures in GRN and other biological networks and can be introduced by data-driven approaches.

The proposed ModularBoost method can be also regarded as a low-weight solution to deal with time-series and single cell expression data. Based on experimental outcomes about curated and scRNA-seq datasets, the ModularBoost method is able to improve inference accuracy as well as to reduce computational time. It can be understood that decomposition of network inference can reduce the computational burden, since an original task was transferred to multiple sub-tasks. The purpose is to obtain topologies with better biophysical or biomedical explanations. To evaluate the effectiveness of identified regulatory modules, relevant annotations called Module gold will be necessary. This study applies module labels and compute $F_{rr}$ index to quantitatively evaluate the performance of data-driven gene module identification.

## ModularBoost methods

### ICA-FDR based gene module identification

Gene module correspond to the group of genes with similar expressive patterns and biological functions. Researches about gene module help researchers better understand disease modules and gene-disease relations.

**Fig. 6:**
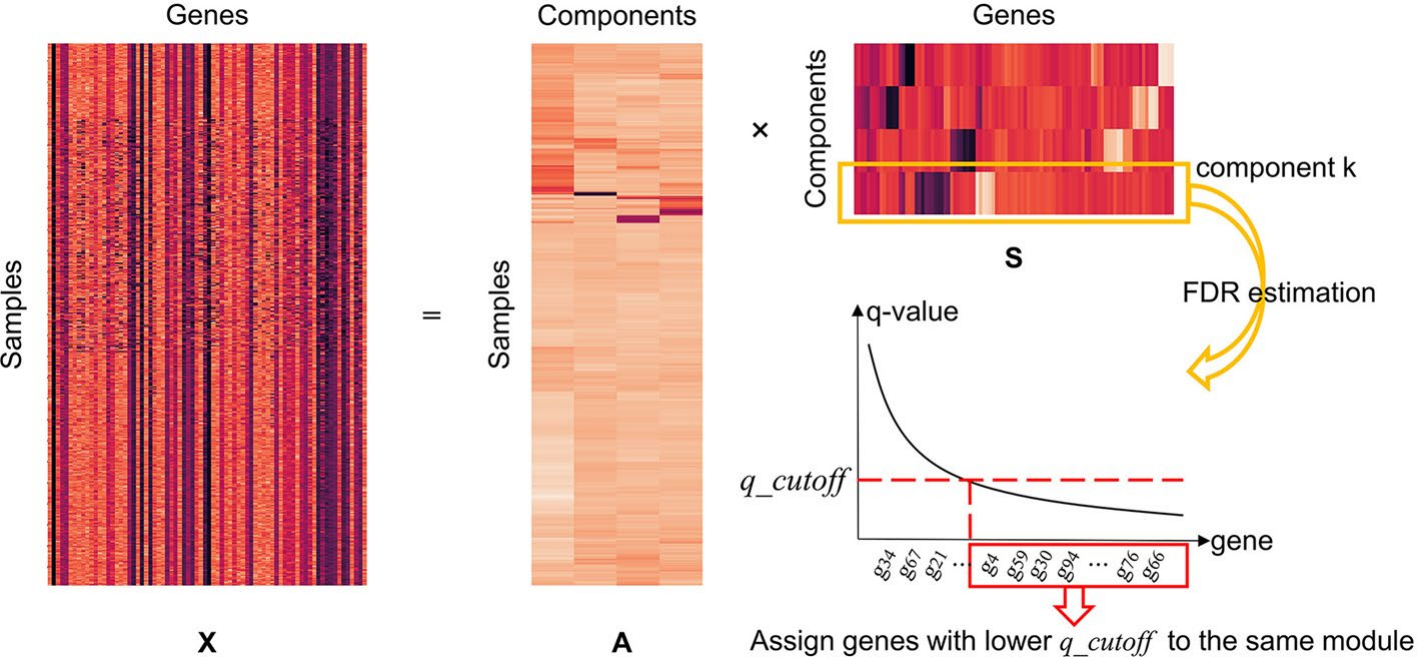
An overview of the ICA-FDR decomposition method. FastICA splits the expression *X* into a mixing matrix *A* and a source matrix *S*. Contained in the rows of *S*, the components reflect hidden biological processes influencing gene expression. The level of genetic influence on components are reflected by the heat color map, from dark (minimum) to red (maximum). FDR estimation determines which genes are assigned to each module

In general, the intent of the independent component analysis (ICA) is to find the hidden ‘independent component’ that refers to the gene module in this research [26]. When applied in the field of gene module detection, ICA usually splits express data matrix *X* into two matrices: a source matrix *S* and a mixing matrix *A*, which means $X = AS$ is shown in Fig. 6.

For single cell expression matrix *X*, this study assumes that the columns and rows of *X* correspond to genes and cell samples respectively. The expression value $x_{ij}$ of gene *j* at sample *i* is shown as

2 $$\begin{aligned} x_{ij} = \sum _{k} a_{ik}s_{kj} \end{aligned}$$

where $a_{ik}$ reflects the contribution of cell sample *i* in component *k* and $s_{kj}$ denotes the contribution of component *k* on gene *j* [16]. ICA-decomposition algorithms are different in adopting optimization standards for component independence. In this study, we chose an efficient ICA algorithm—FastICA. The goal of FastICA is to find an orthogonal rotation of prewhitened data through a fixed-point iteration frame [27]. FastICA iteratively maximizes non-Gaussian of the rotated components until convergence, and *k* independent signals that corresponded *k* gene modules are found in this process.

FastICA algorithm tries to find gene components in the source matrix that own non-Gaussian characteristics. Each modular signal in the source matrix generally obeys a heavy-tailed normal distribution. Under this circumstance, those genes at the tails have a significant contribution to those components, while the majority of genes in peak have weak impact. In the next step, we applied the false discovery rate (FDR) estimation to assign genes to various functional modules.

The basic procedure of the ICA-FDR algorithm is shown as Algorithm 1.

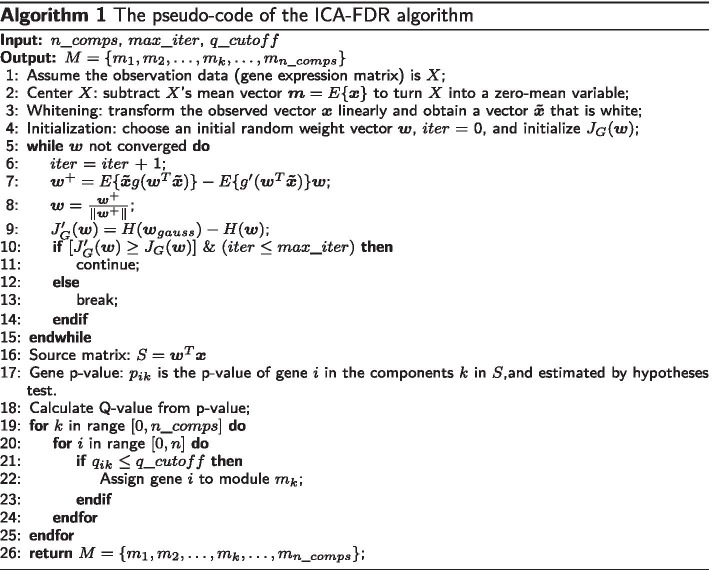



Input parameters include the number of gene modules $n\_comps$, the maximum iterations $max\_iter = 20{,}000$ in this research, and the threshold of Q-value *q_cutoff*. The process of whitening was defined as Eq.3.

3 $$\begin{aligned} \varvec{\tilde{x}} = \varvec{ED}^{-1/2}\varvec{E}^T\varvec{x} \end{aligned}$$

where $\varvec{E}$ denotes the orthogonal matrix of eigenvectors of $E\{\varvec{xx}^T\}$ and $\varvec{D}$ is the diagonal matrix of its eigenvalues. The first goal of ICA-FDR is maximizing non-Gaussianity, and non-Gaussianity is measured by the approximation of negentropy $J_G(\varvec{w})$ given in the Eq.4.

4 $$\begin{aligned} J_G(\varvec{w}) = H(\varvec{w}_{gauss}) - H(\varvec{w}) \end{aligned}$$

The entropy $H(\cdot )$ can be defined as Eq.5 in the ICA-FDR:

5 $$\begin{aligned} J_G(\varvec{w}) = [E\{G(\varvec{w}^T\varvec{\tilde{x}})\}-E\{G(\nu )\}]^2 \end{aligned}$$

where $\upsilon$ is a Gaussian variable of unit variance and zero mean, and $G(\cdot )$ is the non-quadratic function that is used to improve the robustness of estimation, such as:

6 $$\begin{aligned} G_1(u) = \frac{1}{a_1}\log \cosh a_1u, \ \ \ \ G_2(u) = - exp(-u^2/2) \end{aligned}$$

The $g(\cdot )$ that in Algorithm. 1 is the derivatives of the function in Eq.6:

7 $$\begin{aligned} g_1(u) = tanh(a_1u), \ \ \ \ g_2(u) = u\ exp(-u^2/2) \end{aligned}$$

FDR represents the number of false discoveries in an experiment divided by the total number of discoveries, and the discovery is statistical test that provides an acceptance threshold. Using hypotheses tests, this study first evaluated the statistical significance of genes in each modular signal. This yielded a *p* value for genes in each module, and a ranked list in descending order. Correction for multiple testing was performed by calculating a “Q-value” from the *p* values and estimating FDR values [28]. The formula for calculating a Q-value is defined by Eq.8

8 $$\begin{aligned} q_{ik} = \frac{p_{ik}n}{i} \end{aligned}$$

where $p_{ik}$ is the $i^{th}$ smallest *p*- alue out of *n* gene *p* values for the *k* modular signal.

The fundamental principle of post-process is assigning the genes with lower Q-value than *q_cutoff* to a module, and the process was shown in Fig. 6. According to the number of genes and modules, the value of *q_cutoff* was selected from the set $\{10^{-1}, 10^{-2}, \ldots , 10^{-13}\}$.

The ICA-based decomposition also has several derivatives, including ICA-FDR, ICA-FDR2 and ICA-zscore. ICA-FDR2 is similar to ICA-FDR but divides each component into two modules according to the signs of gene regulations, while ICA-zscore replaces FDR indexes with z-scores to detect module from source signals. As a tool to reduce dimension, the principal component analysis (PCA) can be used to visualize the similarities among the biological samples [29]. We tested the performance of these methods in the experiments to compare with the ICA-FDR.

### Decomposition-based GRN hybrid inference

**Fig. 7:**
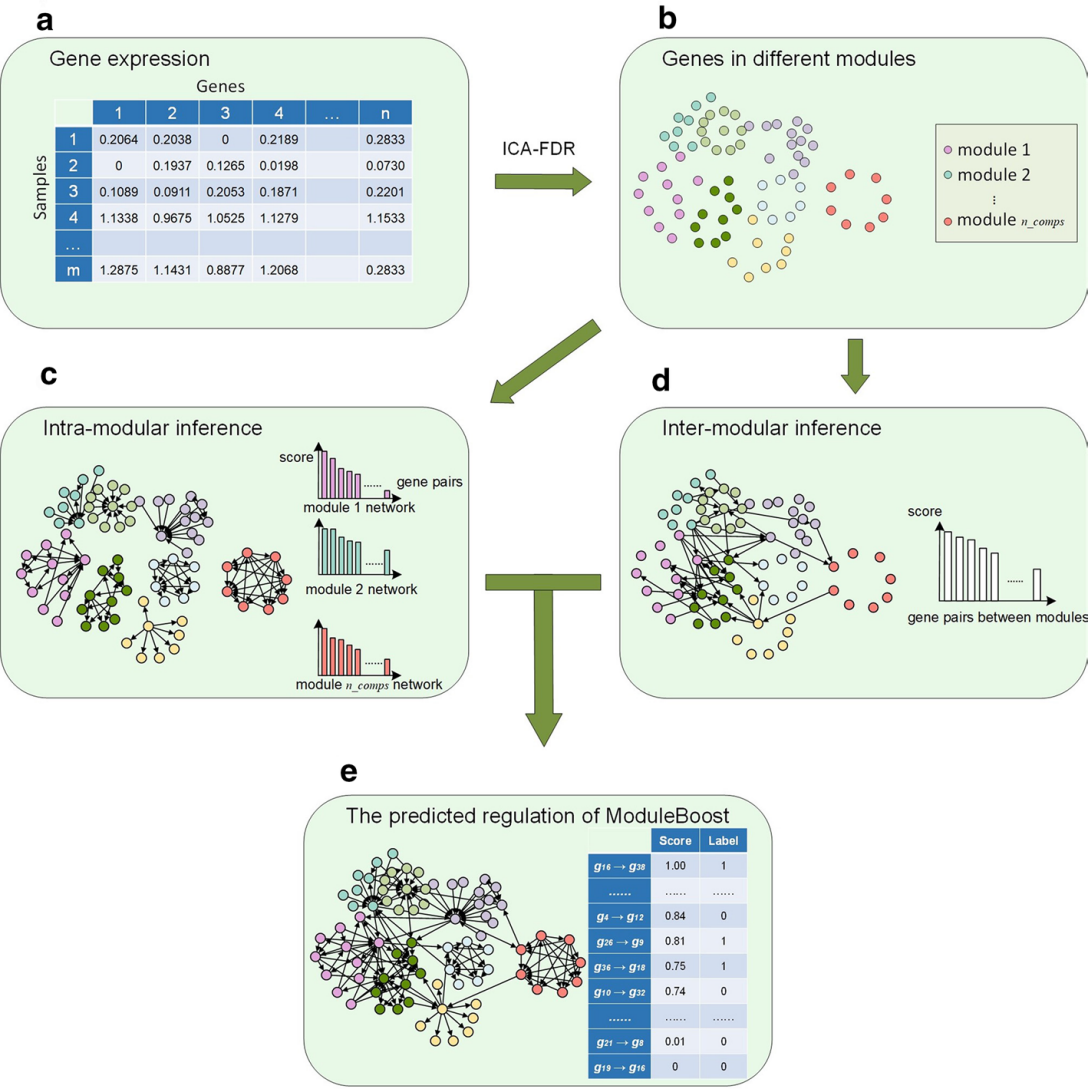
The workflow of ModularBoost. (**a**) Input: time-stamped single-cell gene expression data; (**b**) Step 1: based on expression patterns, ICA-FDR assigns the genes into several modules with various colors; (**c**) Step 2: GRNBoost2 infers GRN for each gene module separately and sorts $n\_comps$ groups of scores in descending order; (**d**) Step 3: the inter-modular interactions are computed by sparse regression; (**e**) Step 4: normalization of inference scores separately, and computation the amalgamated edge predictions of the GRN

Different NI algorithms have their unique characteristics, and the combination of multiple algorithms can provide a possible strategy to obtain networks with sparse and dense interactions [11]. The ICA-FDR algorithm has divided genes into different modules. For GRNs, functionally correlated genes or TFs form gene modules in which the intra-modular connections are tenser than of inter-modular relations. In this section, based on the detected gene modules, we proposed an algorithm that uses GRNBoost2 to infer intra-modular interactions and ridge regression to determine inter-modular regulations, which conforms to community structures. Before calculating the inter-modular connections, the proposed ModularBoost approach removes those gene pairs that are in the same module to reduce computational burden. The workflow of ModuleBoost is shown in Fig. 7.

#### Infer intra-modular interactions using GRNBoost2

Based on a similar concept as GENIE3, GRNBoost2 infers regulators for every target gene purely from the gene expression matrix [4]. The conventional GRNBoost2 inference algorithm was based on Gradient Boosting Machine (GBM) regression that focuses on efficiency and had been a top-ranking algorithm in GRN inference. One character of GRNBoost2 is global estimation of decision tree number with a self-tuning mechanism.

The set of $n\_comps$ modules that decomposed by the ICA-FDR is defined as $M = \{ m_1, m_2, \ldots , m_k, \ldots , m_{n\_comps}\}$, where the $m_k = \{g_1^k, g_2^k, \ldots , g_{k_n}^k\}$, $k = 1,2,\ldots , n\_comps$, and there are $k_n$ genes assigned in $m_k$. In what follows, we applied GRNBoost2 at each module $m_k$ and separately inferred the intra-modular connections. Learning samples can be constructed as $LS^k =\{\mathbf{x }_1, \mathbf{x }_2, \ldots , \mathbf{x }_s, \ldots , \mathbf{x }_N\}$, where *N* is the number of samples or experiments in the gene expression matrix, and $\mathbf{x} _s = (x_s^1, x_s^2, \ldots , x_s^{k_n})^T$ is a vector of genes expression data in *s*th sample.

GRNBoost2 assumes that the expression levels of the genes in $m_k$ can be represented by the other genes in the module with random noise. This indicates that $\mathbf{x} _s^{-j}$ can be defined as the vector of genes except gene *j* in *s*-th observation samples, i.e. $\mathbf{x} _s^{-j} = (x_s^{1}, x_s^{2}, \ldots , x_s^{j-1}, x_s^{j+1}, \ldots , x_s^{k_n})^T$. Therefore, expression behaviors of the target gene *j* are controlled by the other genes, shown by Eq.9:

9 $$\begin{aligned} x_s^j = f_j(\mathbf{x} _s^{-j}) + \varepsilon _s, \ \ \ s = 1,2,\ldots ,N \end{aligned}$$

where $\varepsilon _s$ is a random noise with mean of zero. The function $f_j(\cdot )$ exploits the expression of direct regulators of gene *j*, and it is trained from the learning sample $LS_j^k = \{(\mathbf{x} _s^{-j}, x_s^{j}), s = 1,\ldots ,N\}$. Meanwhile, the feature selection computes the confidence level $w_{ij} (i \ne j)$ for the regulatory edge from gene *i* to gene *j*. For the tree regression-based GRN inference method, the function $f_j(\cdot )$ is determined by an ensemble of decision trees. At each tree node $\phi$, the total reduction of the variance for the output variable is computed as:

10 $$\begin{aligned} I(\phi ) = SVar(S) - S_lVar(S_l) - S_rVar(S_r) \end{aligned}$$

where *S* is the set of samples at the tree node $\phi$, $S_l$ and $S_r$ denote the left and right subtrees, $Var(\cdot )$ represents the variance during splitting.

For GRNBoost2, each decision tree is trained by a random subset of samples that cover approximately 90$\%$ of raw data, and 10$\%$ others are called out-of-bag samples that improve the loss function [5]. Trees stop growing up when the loss function meets the early-stopping rule that the average loss improvement drops to 0.

Therefore, gene modules correspond to subnetworks with multiple topological importance levels. Gathering regulatory associations and sorting in descending order, the intra-modular inference finalizes the output as shown in Fig. 7c.

#### Infer inter-modular connections using sparse regression

The regulatory associations between regulators and target genes in the same module were inferred by ensemble-based GRNBoost2 algorithm, while the relationships controlled by regulators that outside the module are still unknown. The combination of sparse regression algorithms with biologically meaningful constraints may provide a promising solution to enhance accuracy of GRN inference. Based on this hypothesis, we recovered regulatory relations among genes in different modules by adopting regularized linear regression (ridge regression) and used a pre-treatment to reduce computational complexity. The visualization of inter-modular inference is available in Fig. 6d.

In the following, given *n* genes, *m* samples, and the expression matrix $E_{m \times n}$, the linear regression problem can be defined as:

11 $$\begin{aligned} \varvec{E}_t = \alpha _{r_1,t}\varvec{E}_{r_1} + \alpha _{r_2,t}\varvec{E}_{r_2} + \ldots + \alpha _{r_t,t}\varvec{E}_{r_t} + \varvec{\beta }_t \end{aligned}$$

where $\varvec{E}_t$ is a expression value vector of target gene $g_t\in G = \{g_1, g_2, \ldots , g_n\}, t = 1,2, \ldots ,n$. The potential regulators of $g_t$ that not in the same module with $g_t$ are denoted by $G^{-t} = \{g_{r_1}, g_{r_2},\ldots , g_{r_t}\}$, and $\varvec{E}_{r_1}, \varvec{E}_{r_2}, \ldots , \varvec{E}_{r_t}$ are the expression of $G^{-t}$. Detailed representation is:

12 $$\begin{aligned} \left( \begin{array}{c} e_{1,t} \\ e_{2,t} \\ \vdots \\ e_{m,t} \end{array}\right) = \left( \begin{array}{cccc} e_{1,r_1}^t &\quad e_{1,r_2}^t &\quad &\quad e_{1,r_t}^t \\ e_{2,r_1}^t &\quad e_{2,r_2}^t &\quad &\quad e_{2,r_t}^t \\ \vdots &\quad \vdots &\quad \ldots &\quad \vdots \\ e_{m,r_1}^t &\quad e_{m,r_2}^t &\quad &\quad e_{m,r_t}^t \end{array}\right) \left( \begin{array}{c} \alpha _{r_1,t} \\ \alpha _{r_2,t} \\ \vdots \\ \alpha _{r_t,t} \end{array}\right) + \left( \begin{array}{c} \beta _{1,t} \\ \beta _{2,t} \\ \vdots \\ \beta _{m,t} \end{array}\right) \end{aligned}$$

where $\alpha _{r_t,t}$ is a regression coefficient describing the influence of regulator $g_{r_t}$ on the target gene $g_t$, and $\varvec{\beta }$ is a vector of noise in regression. For obtaining the solution vector $\varvec{\alpha }$ of each target gene, the objective function of ridge regression is defined as:

13 $$\begin{aligned} L = \arg \min _{\alpha }\Vert \varvec{E}_t-\varvec{E}_r\varvec{\alpha }\Vert ^2_2 + \frac{1}{2}\Vert \varvec{\alpha }\Vert _2^2 \end{aligned}$$

where the quadratic penalty term $\Vert \varvec{\alpha }\Vert _2^2$ makes the loss function convex and leads to a unique minimum.

After the inference of subnetworks and inter-modular connections, a total of $n \times (n-1)$ causal relations and importance scores were computed. Then the regulatory edge scores of intra-modular and inter-modular relations were standardized using maximum-minimum normalization:

14 $$\begin{aligned} x_i' = \frac{x_i - \min (\mathbf{x} )}{\max (\mathbf{x} )- \min (\mathbf{x} )} \end{aligned}$$

According to the normalized important scores, the regulatory associations are combined and ranked in descending order. Therefore, we can calibrate those associations with the gold standard, as shown in Fig. 7e, and use AUROC index to examine whether the real regulatory relationships enrich at the top of the ranking.

### Evaluation metrics

Evaluation metrics are used to quantitatively evaluate the performance of data-driven module identification methods. As indexes F measure, Rand index, and the normalized mutual information face the problem of detecting overlapping modules [9]. This study selects *Recovery*, *Relevance*, and $F_{rr}$ to evaluate the accuracy of ICA-FDR-based module identification methods for their high accuracy and efficiency in handling overlap. The three indexes are in the range of [0, 1], and reach the value 1 only when the observed module and the known module are exactly equal.

$\hat{M}$ and *M* are the set of known and observed modules. In this first stage, a distance (similarity) matrix is computed by the Jaccard index between two modules sets.

15 $$\begin{aligned} Recovery&= \frac{1}{|\hat{M}|}\sum _{\hat{m}\in \hat{M}} \max _{m \in M} Jaccard(m, \hat{m}) \end{aligned}$$

16 $$\begin{aligned} Relevance&= \frac{1}{|M|}\sum _{m \in M} \max _{\hat{m}\in \hat{M}} Jaccard(m, \hat{m}) \end{aligned}$$

The *Recovery* is used to match known modules with observed modules, and the *Relevance* index reflect the extent to which observed modules match with known modules.

Afterwards, the similarity score $F_{rr}$ is summarized by mapping the known modules to detected ones and vice versa. A score quantifying the false negatives (*Relevance*) is calculated by averaging the similarities of known modules and picking out the best representatives in the detected modules. Another score that is related to false positives (*Recovery*) is computed in a similar style. An asymmetric method for module similarity is given by averging *Relevance* and *Recovery* as shown in Eq. 17

17 $$\begin{aligned} F_{rr} = \frac{2\cdot Recovery \cdot Relevance}{Recovery + Relevance} \end{aligned}$$

This study uses the values of area under receive operator curve (AUROC) and area under precision-recall curves (AUPR) to reveal accuracy levels of the ModularBoost network inference algorithm. ModularBoost outputs a descending list of putative regulatory interactions. Picking only the top *K* edges in this list, we compared them with gold standards to assess the number of false positives (FP), true positives (TP), false negatives (FN), and true negatives (TN). ROC curve shows the trade-off between false positive rate (FPR) and true positive rate (TPR) across different *K* thresholds, while PR describes the relationship between recall and precision. FPR, TPR, recall, and precision are expressed as:

18 $$\begin{aligned} FPR&= \frac{FP}{TN+FP}, \ \ \ \ TPR = \frac{TP}{TP+FN} \end{aligned}$$

19 $$\begin{aligned} recall&= \frac{TP}{TP+FN}, \ \ \ \ precision = \frac{TP}{TP+FP} \end{aligned}$$

Finally, the AUROC and the AUPR are respectively assessed by computing the area under ROC and PR curves.

## Data Availability

The datasets analyzed during the current study are available in the Github repository, https://github.com/cosinalee/ModularBoost.git

## Abbreviations

GRN: Gene regulatory network
NI: Network inference
PPI: Protein–protein interaction
FDR: False discovery rate
GSD: Gonadal sex determination
TF: Transcriptional factor
ICA: Independent component analysis
PCA: Principal component analysis
AUROC: Area under receive operator curve
AUPR: Area under precision-recall curve
scRNA-seq: single cell RNA sequencing
PrE: Primitive endoderm cells
MEF: Mouse embryonic fibroblast cells
DE: Definitive endoderm cells
GBM: Gradient boosting machine
FP: False positives
TP: True positives
FN: False negatives
TN: true negatives
FPR: false positive rate
TPR: True positive rate
